# PD-1^+^ mast cell enhanced by PD-1 blocking therapy associated with resistance to immunotherapy

**DOI:** 10.1007/s00262-022-03282-6

**Published:** 2022-08-26

**Authors:** Jun Li, Gang Peng, Kuikui Zhu, Xiaohua Jie, Yingzhuo Xu, Xinrui Rao, Yunhong Xu, Yunshang Chen, Biyuan Xing, Gang Wu, Liangliang Shi

**Affiliations:** Cancer Center, Union Hospital, Tongji Medical College, Huazhong University of Science and Technology, Wuhan, 430022 China

**Keywords:** PD-1, Mast cell, Immunotherapy, Cromolyn sodium

## Abstract

**Background:**

Programmed cell death protein 1 (PD-1) antibody has been approved for a variety of tumors, but its effective rate is unsatisfactory. New evidence suggests that mast cells are an important component of the tumor microenvironment and are associated with resistance to immunotherapy, but the underlying mechanism is not clear.

**Methods:**

Bioinformatics analysis of patients with melanoma in TCGA-SKCM and GSE91061 was used to determine the prognostic value of mast cells and their association with anti-PD-1 immunotherapy. HMC-1 cells (mast cell line) and bone marrow-derived mast cells (BMMCs) were used to verify the effect of PD-1 antibody and cromolyn sodium in vitro. The mouse subcutaneous melanoma model was used to verify the effect of the PD-1 antibody on mast cells in vivo.

**Results:**

Bioinformatics analysis showed that mast cells were a poor prognostic factor associated with resistance to anti-PD-1 immunotherapy. PD-1 was expressed on the mast cell membrane. The PD-1 antibody promoted the release of histamine and cytokines from mast cells via the PI3K/AKT pathway and calcium signaling pathway. The activation of mast cells induced by PD-1 antibody could be partially inhibited by cromolyn sodium. In vivo, cromolyn sodium increased the efficacy of PD-1 antibody and decreased the infiltration of mast cells and the density of microvessels.

**Conclusion:**

PD-1^+^ mast cell activated by PD-1 antibody plays a negative role in the tumor microenvironment via the enhanced function of releasing histamine and cytokines. Inhibition of mast cell may provide a new solution to solve the low response rate of anti-PD-1 immunotherapy.

**Supplementary Information:**

The online version contains supplementary material available at 10.1007/s00262-022-03282-6.

## Introduction

Cancer immunotherapy by blocking PD-1/PD-L1 checkpoints has completely changed the treatment of a variety of malignant tumors, resulting in a lasting effect that traditional treatments do not have [1]. However, these treatments are ineffective in a large proportion of patients, and some of the initial respondents eventually became insensitive to treatments. The mechanism of primary and acquired resistance to PD-1/PD-L1 blockade therapy has not been clearly explained. The possible reasons are as follows: insufficient tumor immunogenicity leading to insufficient T-cell infiltration, tumor resistance to interferon, T-cell dysfunction, interruption of antigen presentation, and local immunosuppressive factors in the tumor microenvironment [2].

Mast cell plays an important role in the tumor microenvironment, and its roles, except in allergic reactions, are a concern for an increasing number of researchers [3]. Recent studies have shown that tumor-infiltrating mast cells are related to immunotherapy resistance, and removal of mast cells by drugs greatly improves the effect of immunotherapy [4]. Some studies have shown that high mast cell infiltration is associated with tumor progression and poor prognosis, mainly in colon cancer, gastric cancer, and pancreatic cancer [5]. In gastric cancer, mast cells promote immunosuppression and progression of gastric cancer through the TNF-α-PD-L1 pathway [6].

Mast cells cross-talk with a variety of cells in the tumor microenvironment through degranulation and secretion of cytokines. Mast cells secrete several angiogenic factors, such as vascular endothelial growth factor (VEGF), fibroblast growth factor (FGF), angiopoietin-1, heparin, and tumor necrosis factor-α (TNF-α). Mast cells also secrete several chemokines and cytokines, such as interleukin-5, interleukin-6, and TNF-α, to regulate immune function [7].

PD-1 is expressed not only in activated T cells but also in mast cells [8]. However, the function of PD-1 on mast cells is not clear.

In our study, we explored the function of PD-1 on mast cells by blocking PD-1 with antibody and detecting the release of histamine, cytokines, and chemokines. Then, we examined whether inhibiting the overactivation of mast cells can improve the efficacy of anti-PD-1 immunotherapy in a mouse tumor model.

## Materials and methods

### Patients

The transcriptome data and clinical information of 407 patients in TCGA-SKCM were derived from The Cancer Genome Atlas (TCGA, https://www.cancer.gov/tcga). Transcriptome data and clinical information of 51 patients in GSE91061 were derived from Gene Expression Omnibus (GEO, https://www.ncbi.nlm.nih.gov/geo/query/acc.cgi?acc=GSE91061). In addition, the 51 patients with melanoma were treated with an anti-PD-1 antibody (nivolumab).

### CIBERSORT algorithm to calculate the abundance of activated mast cell

CIBERSORTx (https://cibersortx.stanford.edu/index.php) is an analytical tool from the Alizadeh Lab and Newman Lab that was used to impute gene expression profiles and provide an estimation of the relative fractions of 22 kinds of immune cells in a mixed cell population using gene expression data [9]. We call the relative fraction of immune cells the abundance of immune cells. We inputted transcriptome data from TCGA-SKCM and GSE91061 into this analytical tool, and the abundances of 22 kinds of immune cells in each sample were calculated. We extracted the data on the abundance of activated mast cell in 22 kinds of immune cells for follow-up analysis.

### Display of PD-1 expression in mast cell by single-cell sequencing

Tumor Immune Single-cell Hub (TISCH) is a single-cell RNA sequencing database focusing on the tumor microenvironment [10]. We first screened all single-cell sequencing datasets, including mast cell. Next, all cell types and PDCD1 were selected, and the figure generated by the website was downloaded.

### Cell culture

The HMC-1 cells were a generous gift from Professor Liu of Wuhan Tongji Hospital. The mouse melanoma cell Line B16-F10 was purchased from American Type Culture Collection (ATCC). The two cell lines were cultured in 1640 complete medium (10% fetal bovine serum and 1% penicillin/streptomycin) in a 37 °C incubator (5% CO2).

### Induction of bone marrow-derived mast cells

Bone marrow-derived mast cells (BMMCs) were induced from 5-week-old female BALB/c mice. We collected the cells from the femur, lysed the red blood cells, and adjusted the cell density to 5*10^5/ml. The cell medium was 1640 complete medium (10% fetal bovine serum, 1% penicillin/streptomycin, 10 ng/ml IL-3, 20 ng/ml SCF). We cultured the cells for a total of 5 weeks and changed the medium once a week.

### Animal experiments

C57BL/6 mice (5 weeks old, female) were purchased from the Experimental Animal Center of Three Gorges University. After one week of adaptive feeding, 1*10^6 B16-F10 melanoma cells were injected subcutaneously. When the diameter of the tumor was 5–9 mm, the mice were randomly divided into four groups. There were six mice in each group. The control group was given 100 μl PBS, the cromolyn sodium group was given 100 μl 2 mg/ml cromolyn sodium (Sigma, St Louis, MO, USA) every day, the PD-1 antibody group was given 100 μl 2 mg/ml PD-1 antibody (BIOXCELL, New Hampshire, USA) once every 3 days, and the combined group was given 100 μl 2 mg/ml PD-1 antibody once every 3 days and 100 μl 2 mg/ml cromolyn sodium every day. Cromolyn sodium was dissolved in normal saline and administered by intraperitoneal injection [11]. The PD-1 antibody was diluted with a special diluent produced by BIOXCELL and administered by intraperitoneal injection. The long diameter (L) and short diameter (W) of the tumors were measured every two days, and the volume was calculated according to the formula: V = LW^2^/2. The tumors were collected after 15 days.

### Enzyme-linked immunosorbent assay (ELISA)

Histamine, TNF-α, VEGF, TGF-β, and CCL2 were detected by ELISA, following the operation steps of kits made by Ruixinbio (Quan Zhou, China).

### Mast cells were stimulated with PD-1 antibody

HMC-1 cells in the logarithmic growth phase and BMMCs were selected, and the cell density was adjusted to 1*10^6/ml. Cells were added to 24-well plates at 1 ml per well. Different concentrations of PD-1 antibody (0, 1, 10 μg/ml, sintilimab injection for HMC-1 cells, PD-1 antibody used in mice provided by Suzhou Xinda for BMMCs) were added to each well. C48/80 is an inducer of mast cell degranulation and a potent phospholipase C inhibitor [12, 13]. Therefore, C48/80 (4 μg/ml, GlpBio, Montclair, CA, USA) was used as a positive control for degranulation. The culture medium was collected after 1 h and 24 h. The supernatant was centrifuged for 5 min at 1000 rpm, and the supernatant was stored at -80 °C for detection.

### Cromolyn sodium pretreatment

Cromolyn sodium powder (Sigma, St Louis, MO, USA) was diluted to 2 mg/ml with PBS. The mast cells in the logarithmic growth phase were centrifuged and resuspended in Tyrode's salt solution. After being counted by the cell counter, cromolyn sodium solution was added to the treatment group at a concentration of 10 μg/ml, and the same amount of PBS was added to the control group. Cells were placed in a 37 °C incubator for 1 h.

### Toluidine blue staining of mast cells

The tumor tissue of mice was fixed with 4% paraformaldehyde, embedded in paraffin and sliced. The tumor sections were stained with toluidine blue (G1032-100ML, Servicebio) to show mast cells. The cytoplasm of mast cells contains particles composed of heparin and histamine and toluidine blue dyes mast cells with a reddish-purple (heterochromatic) blue background [14]. The number of mast cells and area of each section were counted to obtain the number of mast cells per unit area.

### Immunohistochemistry

The standard method was used for immunohistochemistry. Endothelial cells were labeled with CD34 antibody (1:600, GB11013-1, Servicebio) to show microvessels [15]. Three 400-fold visual fields were randomly selected for each section, the number of microvessels was counted manually, and the average value of the three visual fields was reported. We confirmed whether BMMCs were induced successfully by labeling FcεRI (1:800, GB113166, Servicebio).

### Immunofluorescence

HMC-1 cells were fixed with 4% paraformaldehyde for half an hour. After washing with PBS 3 times, the cells were blocked with 5% BSA for 1 h. In a 4 °C refrigerator, the cells were incubated with PD-1 antibody (1:100, 18106-1-AP, Proteintech) for 1 h. After washing with TBST 3 times, the cells were incubated with cy3-conjugated goat anti-rabbit IgG (1:500, GB11013-1, Servicebio) for 1 h. Then, the cells were washed with TBST 3 times, and the nuclei were dyed with DAPI (G1012, Servicebio) for 10 min. After washing with TBST 3 times, the cells were resuscitated with anti-fluorescence quenching agent (G1401, Servicebio), the cell suspension was dropped onto a glass slide, and then pictures were taken with a laser confocal microscope. For sections of paraffin-embedded mouse tumors, we used chymase antibody (1:800, GB111085, Servicebio) to show mast cells and PD-1 antibody (1:2000, GB11338-1, Servicebio) to show the PD-1 receptor. We confirmed whether BMMCs were induced successfully by labeling with c-kit (1:800, GB11073-2, Servicebio).

### Western blot

The proteins were separated by SDS‒PAGE and then transferred to PVDF membranes. The PVDF membrane was blocked with 5% nonfat milk for one hour. After eluting nonfat milk with TBST, the PVDF membrane was incubated with the following antibodies for 12 h at 4 °C: AKT (1:1000, 10176-2-AP, Proteintech), p-AKT (1:2000, 66444-1-Ig, Proteintech), and GAPDH (1:1500, GB11002, Servicebio). After eluting the antibody with TBST, the membrane was incubated with goat anti-rabbit IgG secondary antibody conjugated with HRP (1:5000, G1213, Servicebio) for 1 h. An ECL chemiluminescence kit was used to detect the protein content.

### Flow cytometry

HMC-1 cells were fixed with 4% paraformaldehyde for half an hour. The cells were washed with PBS three times after removal from 4% paraformaldehyde. Under dark conditions, the cells were incubated with antibodies for half an hour. One group was incubated with PD-1 antibody (1:100, #135225, Biolegend), and the other group was incubated with isotype control antibody (1:100, #400546, Biolegend). The cells were washed with PBS three times and then analyzed.

### RNA-seq and pathway enrichment analysis

We used TRIzol to extract RNA from cells, and Huada company (Shenzhen, China) sequenced and provided a gene expression matrix. Consensus pathway analysis (CPA) is an online platform for pathway enrichment analysis [16]. We used CPA to analyze our gene expression matrix and obtained some significantly enriched pathways.

### Calcium staining

HMC-1 cells were incubated with 5 μM Fluo-3 AM (CS1213, G-CLONE) for 1 h at 37 °C. After washing twice with HBSS, the cells were incubated at 37 °C for 30 min. Then, the cells were fixed with 4% paraformaldehyde and photographed with a fluorescence microscope.

### Data analysis

The results were reported as the mean of at least three independent experiments (mean ± SEM). A t test was used to analyze the difference between the two groups, and the Mann‒Whitney U test was used for nonnormally distributed data. Pearson’s correlation analysis and linear regression analysis were used to evaluate the correlation between the parameters. The cumulative survival time was calculated by the Kaplan‒Meier method, and the log-rank test was used to compare the two groups. All statistical analyses were carried out by SPSS (version 26.0) software. All the data were tested by a double-tailed test, and *p* < 0.05 was considered to be statistically significant.

## Results

### Activated mast cell is associated with poor prognosis and immunotherapy resistance in melanoma

**Fig. 1:**
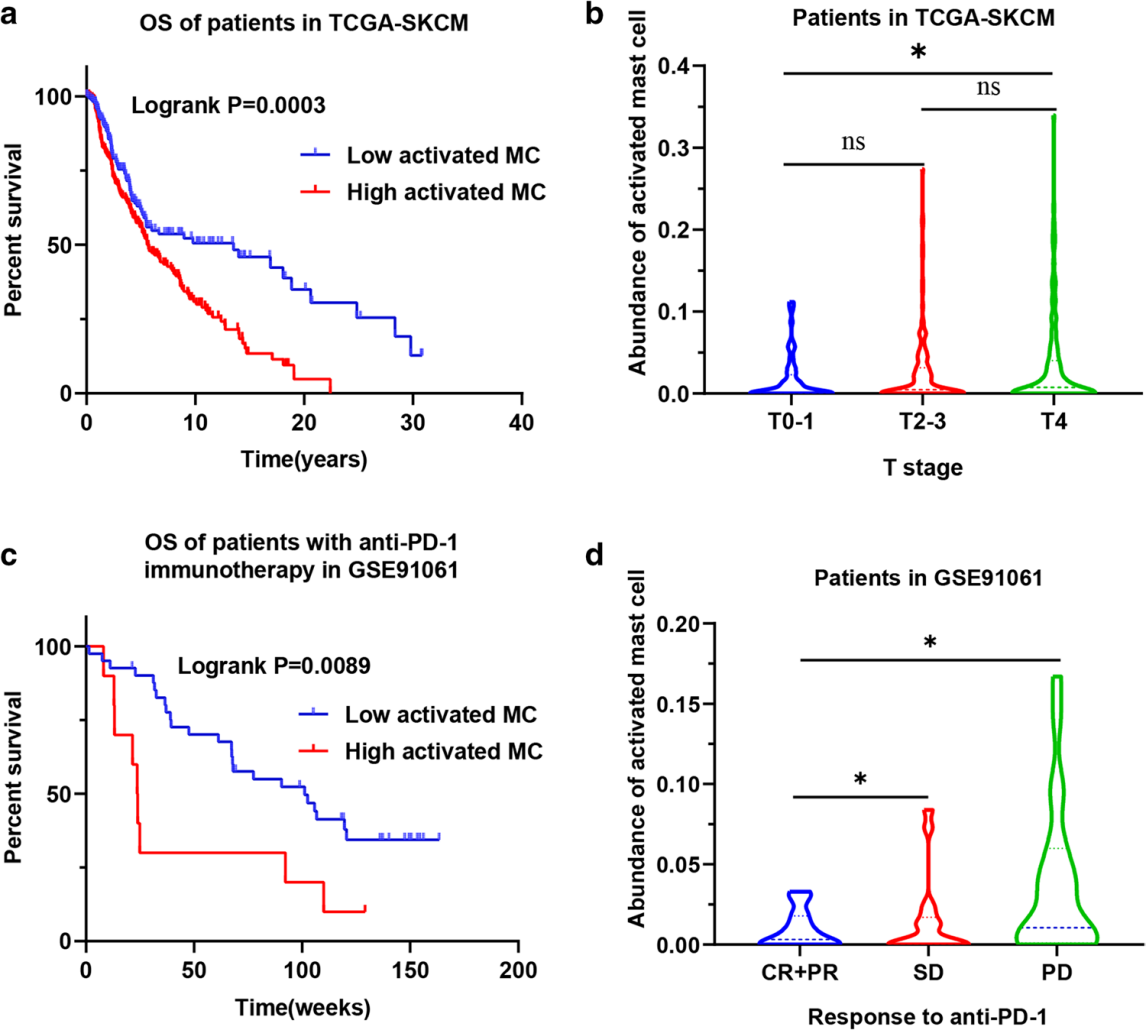
The abundance of activated mast cell is related to the prognosis, T stage, the benefit of immunotherapy, and response to anti-PD-1immunotherapy in melanoma. **a** The abundance of activated mast cell is related to the prognosis of melanoma patients in TCGA-SKCM. **b** The abundance of activated mast cell is related to the T stage of melanoma. **c** The abundance of activated mast cell is related to the effect of anti-PD-1 immunotherapy in patients with melanoma in GSE91061. **d** The abundance of activated mast cell is related to response to anti-PD-1 immunotherapy. *p < 0.05. CR complete response, PR partial response, SD stable disease, PD progressive disease

To evaluate the potential role of mast cell in melanoma, we analyzed the clinical information, prognostic information, and transcriptome data of 407 patients with melanoma in TCGA-SKCM and 51 patients with melanoma in GSE91061. The CIBERSORT algorithm was used to process the transcriptome data to calculate the abundance of activated mast cell in each tumor sample. In TCGA-SKCM, it is worth noting that the abundance of activated mast cell in tumors is related to the prognosis of patients, and patients with a low abundance of activated mast cell have a better prognosis (Fig. 1a). Next, we compared the abundance of activated mast cell in patients with different T stages (Fig. 1b). We found that a high abundance of activated mast cell was associated with a high T stage, and the abundance of activated mast cell in T4 patients was significantly higher than that in T0-1 patients. However, no correlation was found between the abundance of activated mast cell and the N stage or M stage (Supplementary Fig. 1a, b).

To explore the role of activated mast cells in anti-PD-1 immunotherapy, 51 melanoma patients who were treated with PD-1 antibody in GSE91061 were analyzed. First, we analyzed the prognosis of patients with a different abundance of activated mast cells and found that patients with a low abundance of activated mast cells had a better prognosis (Fig. 1c). The benefits of immunotherapy are directly related to the response to treatment, so we explored the abundance of mast cells in tumors of patients with different treatment responses. The results showed that a higher abundance of mast cells was associated with a worse treatment response (Fig. 1d). Taken together, these findings suggest that mast cell activation is associated with poor prognosis and immunotherapy resistance in melanoma.

### Mast cells express PD-1

To determine the expression of PD-1 in mast cells, we first analyzed the single-cell sequencing data of various tumors. As shown in Supplementary Fig. 2, mast cells can be detected in a variety of tumors. Then, the average expression of the programmed cell death 1 (PDCD1) gene at the RNA level of mast cells and other immune cells in different datasets was calculated, and the results showed that mast cells constitutively expressed PDCD1, which was much lower than that of CD8 + T cells. In addition, mast cells and PD-1 in tumor sections of mice were labeled by immunofluorescence, and the results showed that mast cells expressed PD-1 in tumors (Supplementary Fig. 3).

**Fig. 2:**
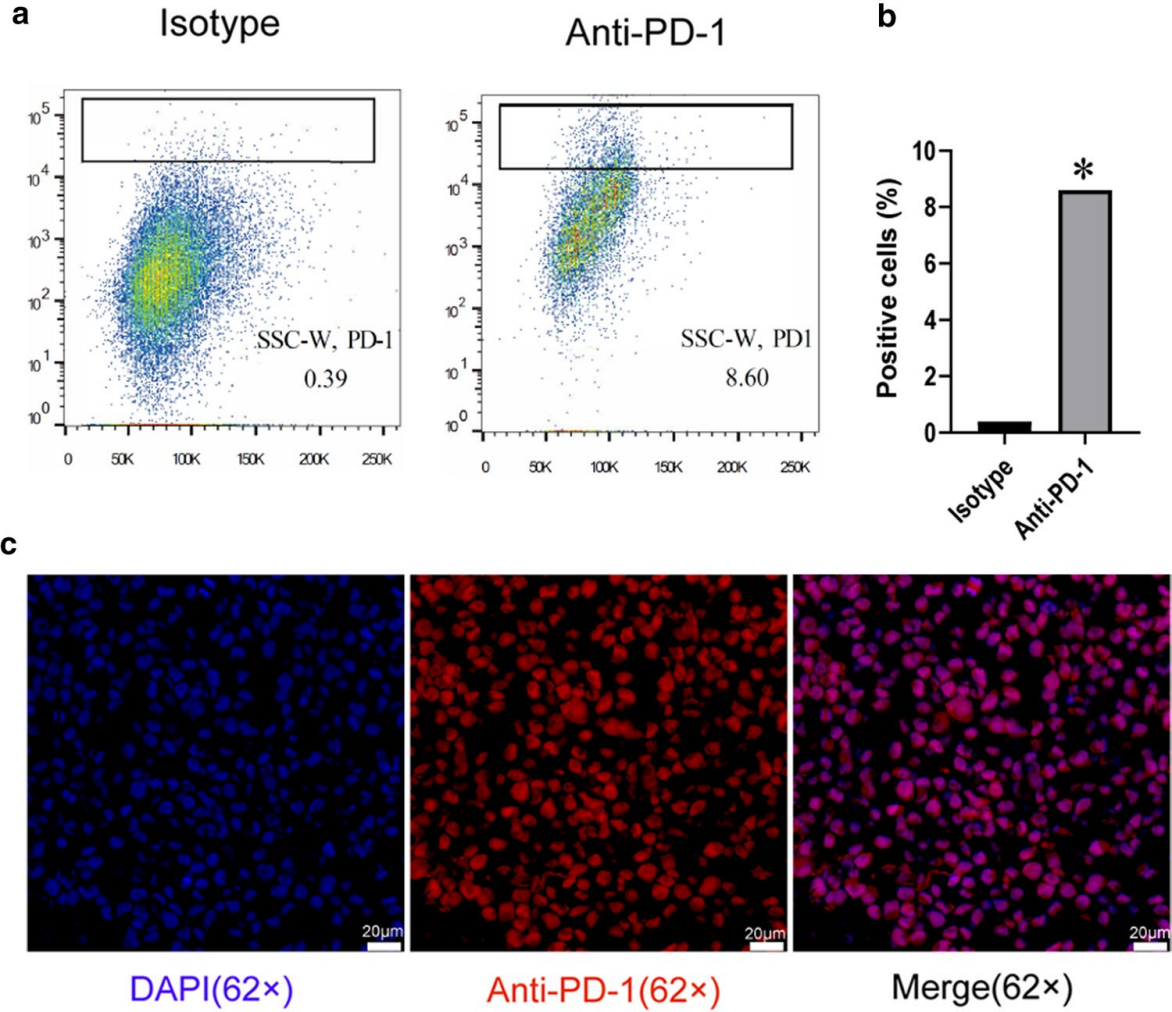
Mast cells express PD-1. **a** Flow cytometry showed that mast cells express PD-1 receptor. **b** Statistical diagram of positive cells by flow cytometry. **c** Immunofluorescence showed that mast cells express PD-1 receptor

HMC-1 is a widely used human mast cell line that has many similar characteristics to mast cells in vivo. We used flow cytometry (Fig. 2a, b) and immunofluorescence (Fig. 2c) to detect PD-1 on the surface of HMC-1 cells to prove the presence of PD-1 on the surface of mast cells. We induced bone marrow-derived mast cells (BMMCs), and the expression of FcεRI and c-kit (more than 95%) proved that the induction was successful (Supplementary Fig. 4, 5). PD-1 on the surface of BMMCs was detected by immunofluorescence (Supplementary Fig. 6). The above results were consistent with the analysis of the RNA expression of PD-1 in mast cells, indicating that there is PD-1 on the membrane of mast cells.

### PD-1 antibody enhanced the function of mast cell in the secretion of histamine and cytokines

**Fig. 3:**
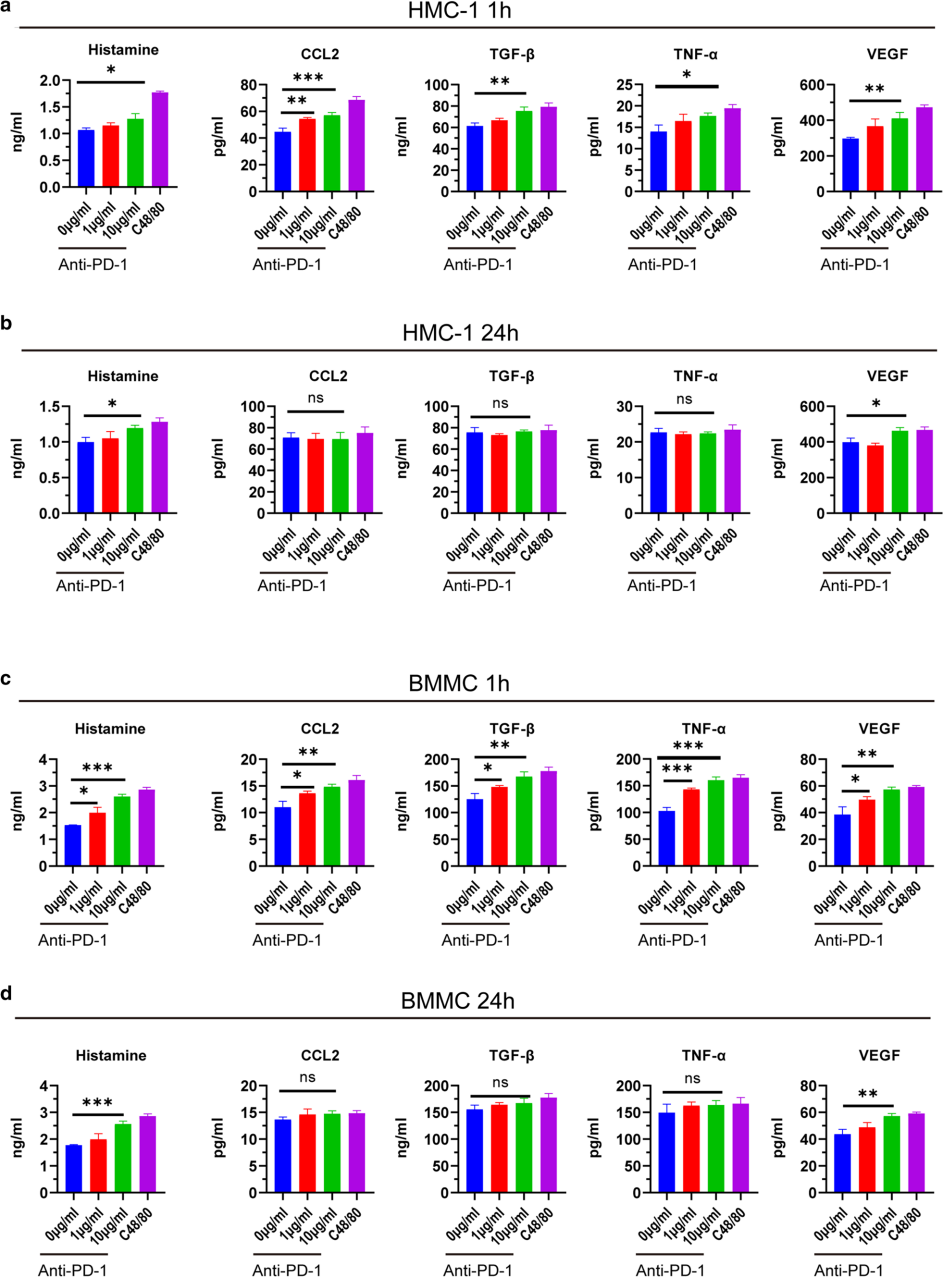
PD-1 antibody promoted secretion of histamine and cytokines of mast cell. **a** Secretion of histamine and cytokines after being treated by PD-1 antibody (1 μg/ml,10 μg/ml) for 1 h in HMC-1. **b** Secretion of histamine and cytokines after being treated by PD-1 antibody (1 μg/ml,10 μg/ml) for 24 h in HMC-1. **c** Secretion of histamine and cytokines after being treated by PD-1 antibody (1 μg/ml,10 μg/ml) for 1 h in BMMC. **d** Secretion of histamine and cytokines after being treated by PD-1 antibody (1 μg/ml,10 μg/ml) for 24 h in BMMC. *p < 0.05, **p < 0.01, ***p < 0.001

We studied whether PD-1 antibody enhances the function of mast cell. First, HMC-1 and BMMC mast cells were treated with different concentrations of PD-1 antibody (0, 1, 10 μg/ml), and C48/80 was used as a positive control. The supernatants of mast cells were taken at different time points (1 h, 24 h), and the concentrations of histamine, TNF-α, VEGF, CCL2, and TGF-β were detected by ELISA. The results showed that the PD-1 antibody promoted the release of the above substances at 1 h after treatment with anti-PD-1 (Fig. 3a, c). After 24 h of treatment, we observed the promoting effect of PD-1 antibody on histamine and VEGF (Fig. 3b, d).

### PD-1 antibody regulated the function of mast cell by activating the PI3K/AKT pathway and calcium signaling pathway

**Fig. 4:**
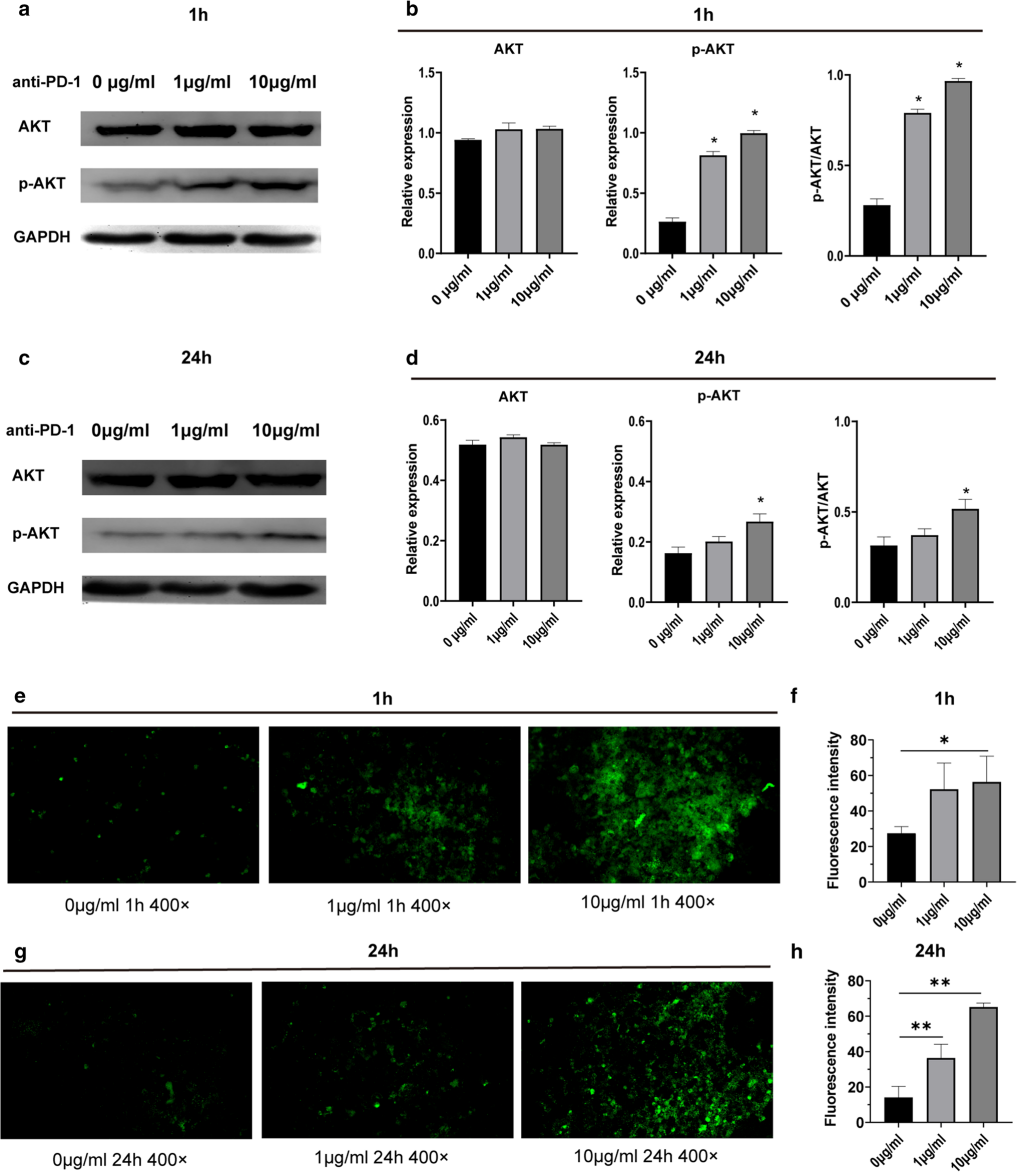
PD-1 antibody regulated function of HMC-1 through PI3K/AKT pathway and calcium signaling pathway. **a** Western blotting of PI3K/AKT related protein expression after treated by PD-1 antibody (1 μg/ml,10 μg/ml) for 1 h. **b** Relative expression of AKT, p-AKT to GAPDH after treated by PD-1 antibody (1 μg/ml,10 μg/ml) for 1 h. **c** Western blot of PI3K/AKT related protein expression after treated by PD-1 antibody (1 μg/ml,10 μg/ml) for 24 h. **d** Relative expression of AKT, p-AKT to GAPDH after treated by PD-1 antibody (1 μg/ml,10 μg/ml) for 24 h. **e** Calcium staining of mast cells treated by PD-1 antibody (1 μg/ml,10 μg/ml) for 1 h. **f** Fluorescence intensity of calcium in mast cells treated by PD-1 antibody (1 μg/ml,10 μg/ml) for 1 h. **g** Calcium staining of mast cells treated by PD-1 antibody (1 μg/ml,10 μg/ml) for 24 h. **h** Fluorescence intensity of calcium in mast cells treated by PD-1 antibody (1 μg/ml,10 μg/ml) for 24 h. *p < 0.05, **p < 0.01

To study the mechanism of mast cell activation by PD-1 antibody, we searched the mast cell activation pathway and PD-1/PD-L1 pathway in the KEGG database. In T cells, PD-1 antibody activates the PI3K/AKT pathway, MAPK/ERK1/2 pathway, calcium signaling pathway and so on. Interestingly, mast cells can be activated by allergens via the PI3K/AKT pathway and calcium signaling pathway [17]. We sequenced the RNA of HMC-1 cells treated with or without PD-1 antibody (10 μg/ml, 24 h), and pathway enrichment analysis was carried out. We found enrichment of the PI3K/AKT pathway and calcium signaling pathway, but we did not find enrichment of the MAPK/ERK1/2 pathway (Table 1). Therefore, we tested the PI3K/AKT pathway by western blotting and the calcium signaling pathway by calcium staining. Mast cells were treated with PD-1 antibody (0 μg/ml, 1 μg/ml and 10 μg/ml) for 1 h and 24 h. Compared with untreated mast cells, p-AKT increased significantly (Fig. 4a–d), and the calcium concentration increased significantly (Fig. 4e–h). In short, PD-1 antibody may activate mast cells through the PI3K/AKT pathway and calcium signaling pathway.

**Table 1:** enrichment analysis of pathways

Pathway	Wilcox-FDR	KS-FDR
Calcium signaling pathway	2.12E-11	8.87E-10
PI3K/AKT pathway	0.043213751	0.023111446
MAPK/ERK1/2 pathway	0.944227272	0.938490981

### Cromolyn sodium inhibited the release of histamine and cytokines

**Fig. 5:**
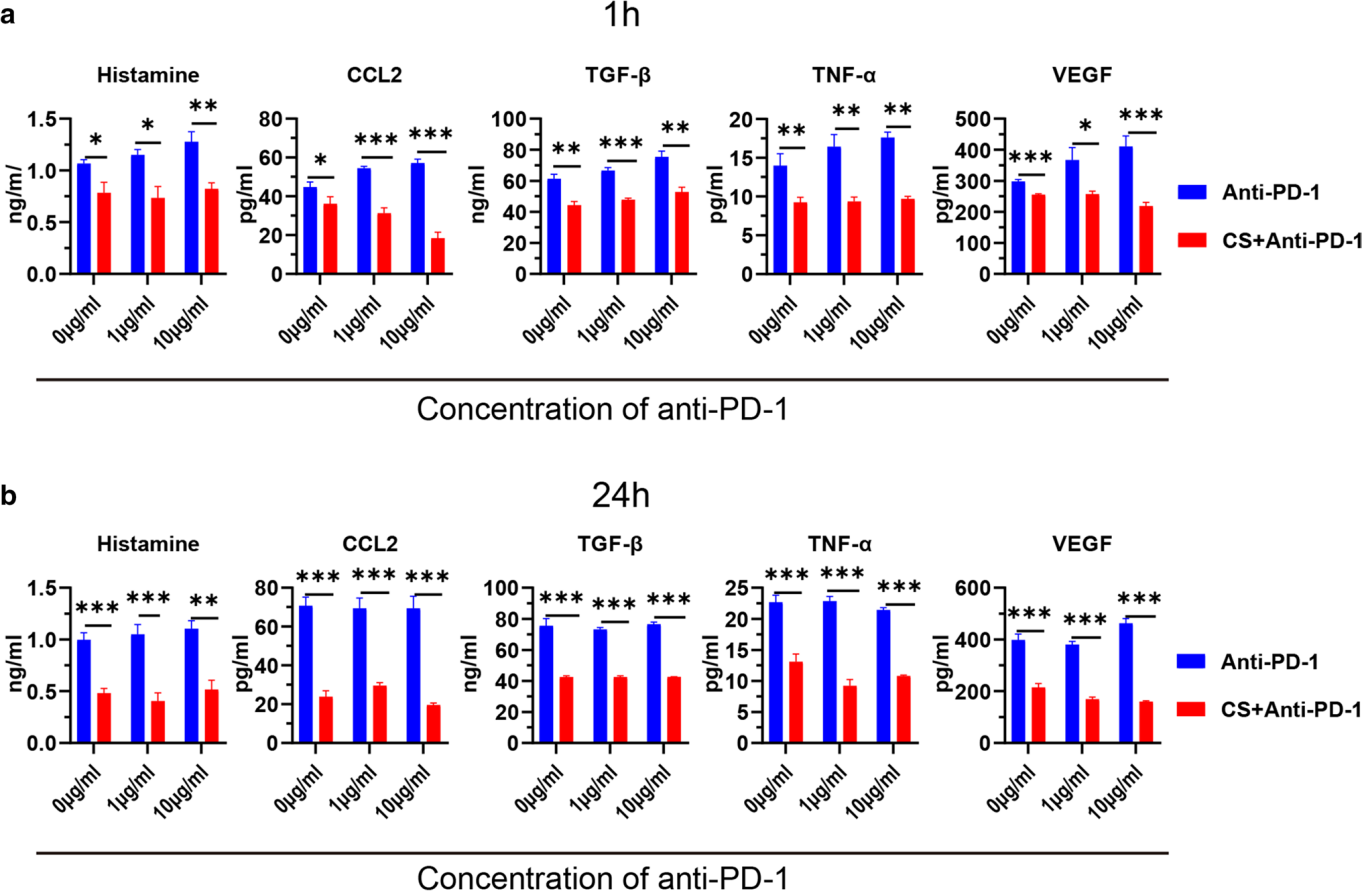
Cromolyn sodium inhibited degranulation and releasing cytokines of mast cells. a Cromolyn sodium inhibited degranulation and releasing cytokines of mast cells after treatment with PD-1 antibody (1 μg/ml, 10 μg/ml) for 1 h. b Cromolyn sodium inhibited degranulation and releasing cytokines of mast cells after treatment with PD-1 antibody (1 μg/ml, 10 μg/ml) for 24 h. CS Cromolyn sodium. *p < 0.05, **p < 0.01, ***p < 0.001

Cromolyn sodium is a classic stabilizer of the mast cell membrane that can inhibit degranulation and release cytokines from mast cells. We studied whether cromolyn sodium can inhibit the function of mast cells when treated with an anti-PD-1 antibody. HMC-1 mast cells were pretreated with cromolyn sodium for 1 h and then incubated with different concentrations of antibodies (1 μg/ml, 10 μg/ml) for 1 h and 24 h. The results showed that cromolyn sodium can inhibit the function of mast cells when treated with PD-1 antibody (Fig. 5a, b).

### Cromolyn sodium improved the efficacy of PD-1 immunotherapy in vivo

**Fig. 6:**
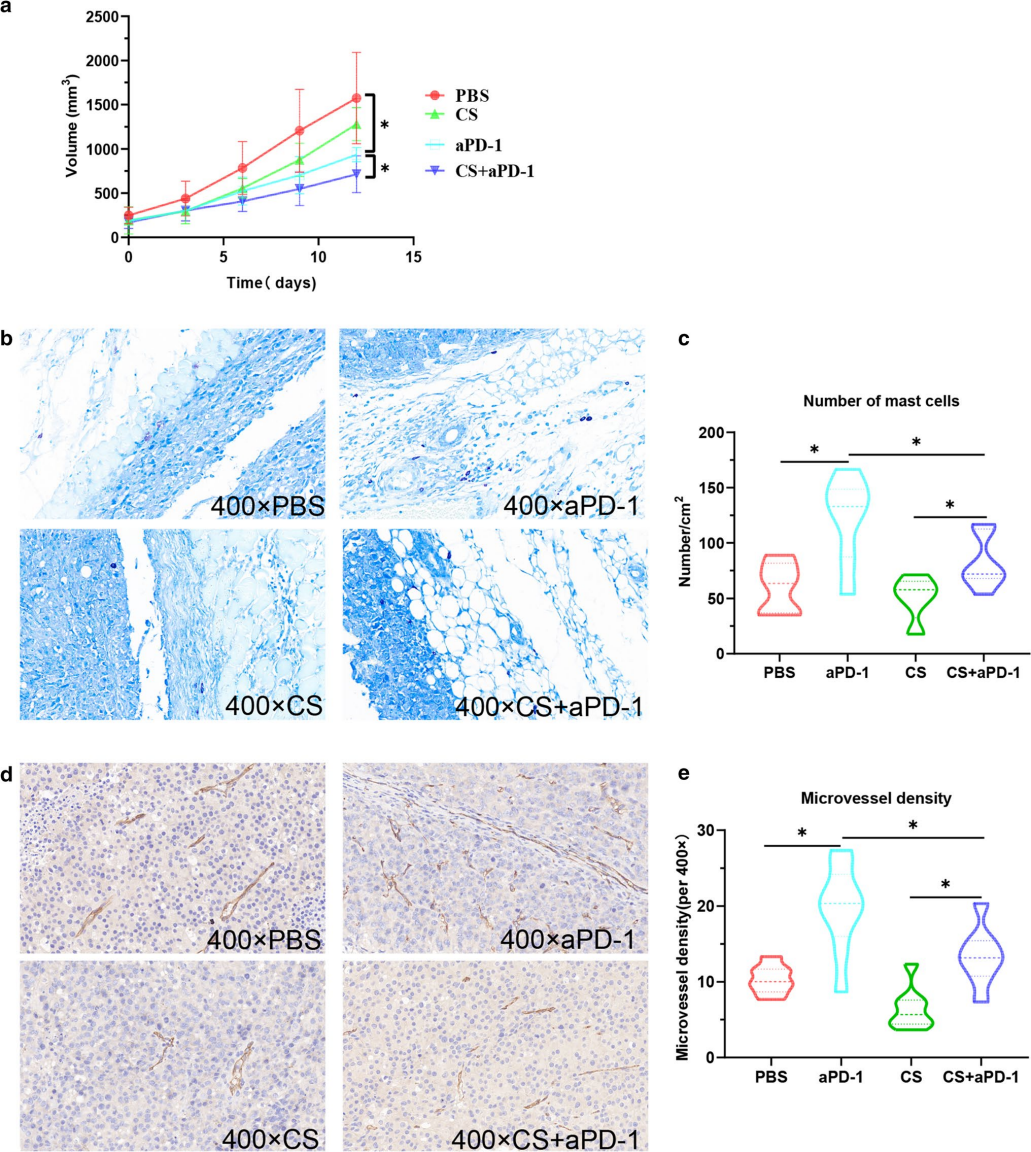
Cromolyn sodium improved the efficacy of PD-1 immunotherapy by inhibiting mast cell. a Growth of tumors in different treatment groups. b Tumor sections were stained with toluidine blue to show mast cells. c The number of mast cells in unit area. d CD34 labeled microvessels in tumor sections. e Microvessels density in tumor sections. CS Cromolyn sodium *p < 0.05

To verify whether cromolyn sodium inhibits degranulation and the release of cytokines from mast cells is beneficial to immunotherapy, we carried out the following experiments on a subcutaneous melanoma tumor model in C57BL/6 mice. When the diameter of tumors was 5–9 mm, the mice were randomly divided into 4 groups and given PBS, cromolyn sodium, PD-1 antibody, or PD-1 antibody combined with cromolyn sodium through subcutaneous injection. The volume of tumors increased the slowest in the PD-1 antibody combined with cromolyn sodium group. Compared with the PD-1 antibody group, the volume of tumors in the PD-1 antibody combined with cromolyn sodium group was significantly smaller (*p* < 0.05, Fig. 6a, Supplementary Fig. 7).

The tumor sections of mice were stained with toluidine blue to show the number of mast cells in each section (mast cells with reddish-purple). Compared with the control group (PBS), the PD-1 antibody significantly increased the number of mast cells in the tumors (Fig. 6b, c). Compared with the PD-1 antibody group, the number of mast cells was partially inhibited by cromolyn sodium in the PD-1 antibody combined with cromolyn sodium group (Fig. 6b, c). In addition, we analyzed the activation level of mast cells in different groups by calculating the percentage of degranulated mast cells. We found that PD-1 antibody could increase the percentage of degranulated mast cells, while cromolyn sodium could reduce the percentage of degranulated mast cells (Supplementary Fig. 8).

We confirmed that PD-1 antibody can promote mast cells to secrete VEGF, a cytokine that promotes angiogenesis. Next, we tested whether the PD-1 antibody could increase angiogenesis in the tumor. We detected blood vessels using the immunohistochemical labeling of CD34. Figure 6d, e demonstrate that PD-1 antibody significantly increased microvessel density in the tumor, which could be partially inhibited by cromolyn sodium.

## Discussion

PD-1 antibody can block the PD-1/PD-L1 immune checkpoint and activate T cells, which has been approved for immunotherapy of a variety of tumors [1]. However, in most tumor species, the overall response rate of PD-1 antibodies to unselected patients is only 10–25% [18]. The mechanism of immunotherapy resistance is not clear. Our study suggested a possible mechanism: in the tumor microenvironment, PD-1^+^ mast cells are enhanced by PD-1 antibody, releasing more histamine and cytokines to reduce the effect of immunotherapy.

Mast cell plays an important role in the tumor microenvironment. Somasundaram et al. found that tumor-infiltrating mast cells interact with T_reg_ and downregulate the MHC-I molecule of tumor cells, which is related to immunotherapy resistance, and the removal of mast cells can improve the therapeutic effect [4]. The infiltration of mast cells is associated with a higher stage and poor prognosis in gastric cancer [19]. For patients with systemic mast cell activation syndrome, abnormal continuous activation of mast cells in vivo significantly increased the prevalence of melanoma, breast cancer, cervical cancer, ovarian cancer, lung cancer, and thyroid cancer [20]. In our study, we analyzed the relationship between activated mast cells and prognosis in patients with melanoma in TCGA-SKCM and found that activated mast cells were associated with poor prognosis. For patients who were treated with anti-PD-1 immunotherapy in GSE91061, activated mast cells were associated with poor prognosis, which indicated that mast cells may be involved in immunotherapy resistance.

The PD-1 receptor has an inhibitory effect on mast cells, similar to CD8 + T cells. PD-1 is a receptor that transmits inhibitory signals expressed in CD8 + T cells, Tregs, CD4 + T cells, macrophages, and mast cells [21, 22]. In recent years, studies have gradually revealed the function of PD-1 on cells other than CD8 + T cells. The expression of PD-1 on tumor-associated macrophages suppresses phagocytosis and promotes the formation of the immunosuppressive microenvironment [23]. PD-1 in T_reg_ can reduce the function of immunosuppression and can be reactivated by PD-1 blockade immunotherapy. Once the activation effect of T_reg_ is greater than that of CD8 + T cell, tumor hyperprogression will occur [24]. In our study, we used the HMC-1 mast cell line as the research object to replace mast cell. HMC-1 was proven to express PD-1 by immunofluorescence and flow cytometry, and then PD-1 antibody was used to study the function of PD-1 in HMC-1 cells. Honjo Tasuku elucidated the negative regulation of PD-L1/PD-1 on T cells [25]. Chen's research showed that the binding of PD-L1 in the microenvironment and PD-1 in T cells can induce apoptosis [26]. We hypothesized that the binding of PD-L1 in the microenvironment and PD-1 in mast cells transmits inhibitory signals. Our results showed that PD-1 of HMC-1 cells played an inhibitory role similar to that in T cells, and PD-1 antibody blocking could relieve the inhibition and promote degranulation (increased concentrations of histamine, VEGF, CCL2, TNF-α and TGF-β at 1 h).

We noted that the PD-1 antibody promoted the degranulation of mast cells and caused them to secrete more VEGF. It is well known that vascular abnormalities are the hallmark of most solid tumors and contribute to immune escape, while VEGF is closely related to angiogenesis [27]. VEGF can also disable CD8 + T cells by inducing the expression of TOX [28]. It is believed that the combination of antiangiogenic therapy and immunotherapy may increase the effectiveness of immunotherapy and reduce the risk of immune-related adverse reactions. Through the immunohistochemical analysis of tumors in mice after immunotherapy, we found that PD-1 antibody increased tumor microvessel density compared with the control group. This suggested that mast cells activated by PD-1 antibody release more VEGF to promote angiogenesis, which may be one of the mechanisms by which mast cells participate in immunotherapy resistance.

In addition to VEGF, PD-1 antibody promoted mast cells to release some substances, including TNF-α, TGF-β, and CCL2. Mast cells can secrete TGF-β to promote the function of T_reg_ [29]. In hepatocellular carcinoma, the CCL2/CCR2 axis promotes macrophage infiltration and forms an immunosuppressive microenvironment [30]. Mast cells release TNF-α near blood vessels to promote the infiltration of neutrophils [31], while some subsets of neutrophils can inhibit the activation of T cells [32]. Our results showed that PD-1 antibody can promote mast cells to communicate with other cells, which is a potential mechanism by which mast cells affect the effectiveness of immunotherapy.

Cromolyn sodium can inhibit the function of mast cells and increase the efficacy of immunotherapy. Cromolyn sodium, a classic stabilizer of the mast cell membrane, can inhibit degranulation [33]. We confirmed that the enhancement effect of PD-1 antibody on mast cells could be partially weakened by pretreatment with 10 μg/ml cromolyn sodium for 1 h before treatment with PD-1 antibody. In thyroid cancer, cromolyn sodium can inhibit the tumor-promoting effect of mast cells [34]. Next, in a mouse subcutaneous melanoma model, we verified that cromolyn sodium inhibited the activation of mast cells treated with PD-1 antibody in vivo and then increased the efficacy of immunotherapy. The growth data showed that cromolyn sodium can enhance the efficacy of PD-1 antibody, and cromolyn sodium alone tends to inhibit tumors. Motawi's study showed that cromolyn sodium can directly inhibit the growth of tumor cells in vitro, which suggests that cromolyn sodium alone may inhibit tumors [35]. However, in our in vivo experiments, cromolyn sodium alone did not significantly inhibit tumors, which may be related to the dose of cromolyn sodium. PD-1 antibody is a drug approved for clinical use, so PD-1 antibody alone is also effective, consistent with our results. Our prognostic analysis suggested that mast cells play a tumor-promoting role in PD-1 immunotherapy for melanoma. When cromolyn sodium is combined with PD-1 antibody, cromolyn sodium can inhibit the tumor-promoting effect of mast cells, so this treatment inhibited tumor growth to the greatest extent. To show the infiltration of mast cells, the tumor sections were stained with toluidine blue. Then, we found that PD-1 antibody significantly increased the number of mast cells in the tumors and that cromolyn sodium reduced the number of mast cells. Song et al. have shown that the activation of the PI3K pathway can promote the migration of mast cells [36]. Our results showed that the PD-1 antibody activated the PI3K pathway. Using CD34 antibody to label vascular endothelial cells, the results showed that the use of cromolyn sodium could reduce the microvessel density. However, the mechanism of the effect of cromolyn sodium on microvessel density needs to be further studied.

The human immune system is very complex, and there are many kinds of immune cells. The representative cells of adaptive immunity are T cells and B cells, and the representative cells of innate immunity include mast cells, macrophages, dendritic cells, NK cells, eosinophils, and basophils. Mast cells are only part of the body's immune system. Our study only confirmed that mast cells may affect immunotherapy, and PD-1 antibody may also affect clinical outcome by affecting other immune cells, which needs to be further confirmed by a large number of studies. However, our study confirmed for the first time that PD-1 immunotherapy resistance may be attributed to activation of mast cells by PD-1 antibody, which provides a new direction for future immunotherapy research.

In summary, we proposed a new mechanism of resistance to PD-1 immunotherapy: PD-1 antibody not only activates T cells but also activates PD-1^+^ mast cells through the PI3K/AKT pathway. Then, mast cells release more tumor-promoting cytokines and promote tumor growth. Cromolyn sodium can inhibit the promoting effect of PD-1 antibody on mast cell degranulation in vivo and in vitro, thus inhibiting the negative effect of mast cells in the tumor microenvironment and improving the efficacy of immunotherapy.

## Supplementary Information

Below is the link to the electronic supplementary material.

Supplementary file1 (PDF 502 kb)
