# Supplementary material for: PD-1+ mast cell enhanced by PD-1 blocking therapy associated with resistance to immunotherapy
Source: Cancer Immunol Immunother. 2022 Aug 26;72(3):633–45. doi: 10.1007/s00262-022-03282-6 (PMC9947072; doi:10.1007/s00262-022-03282-6)
Supplement: Supplementary file 1 — Supplementary file1 (PDF 502 kb) [file 262_2022_3282_MOESM1_ESM.pdf]

Supplementary Material

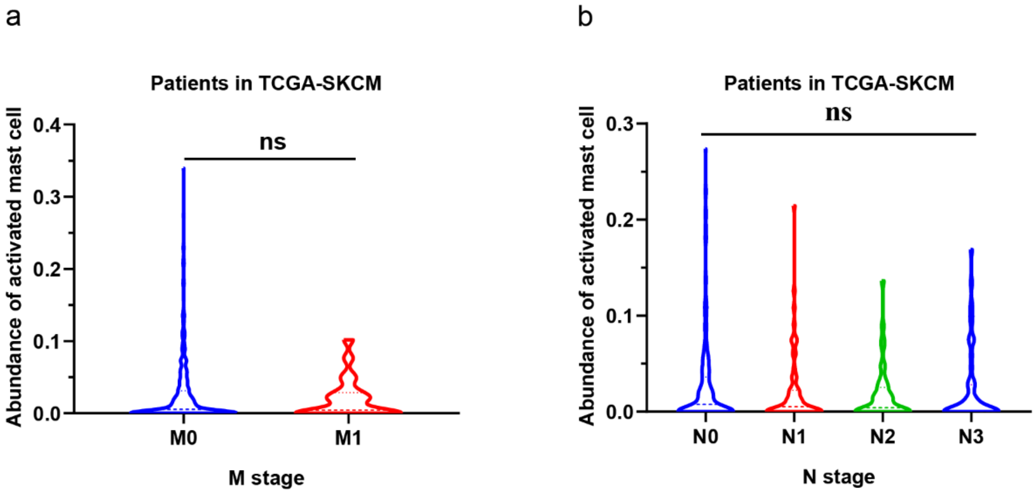

Supplementary Fig. 1 a The relationship of the number of activated mast cells and the M stage of melanoma. **b** The relationship of the number of activated mast cells and the N stage of melanoma.

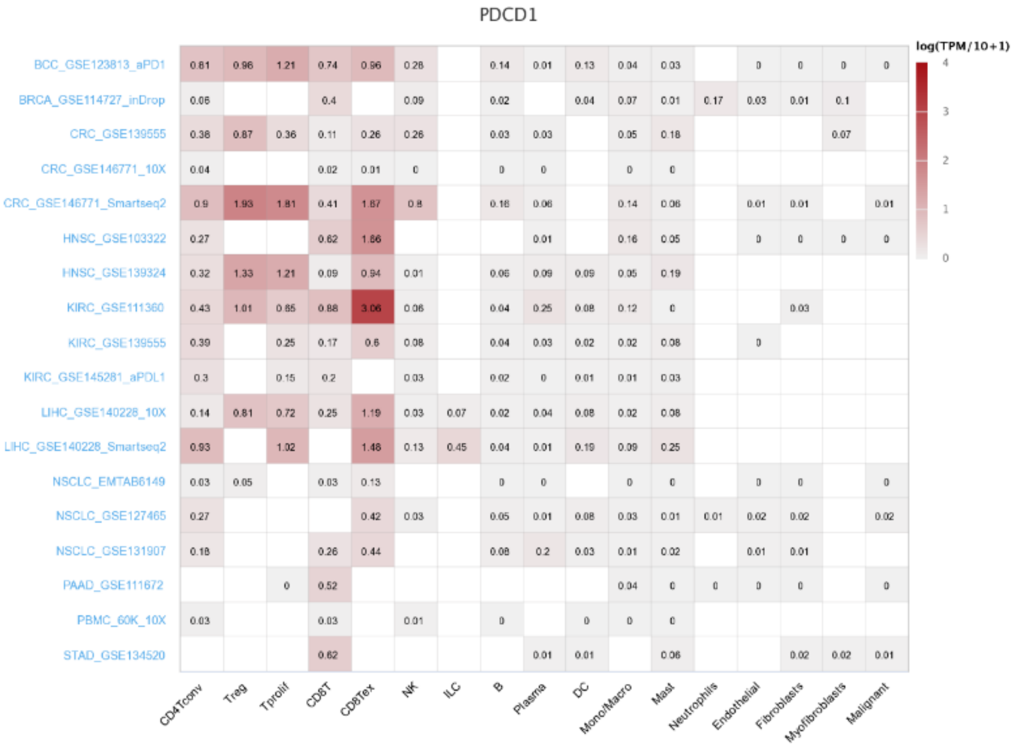

Supplementary Fig. 2 PDCD1 expression of mast cells and other immune cells in different single cell sequencing data sets.

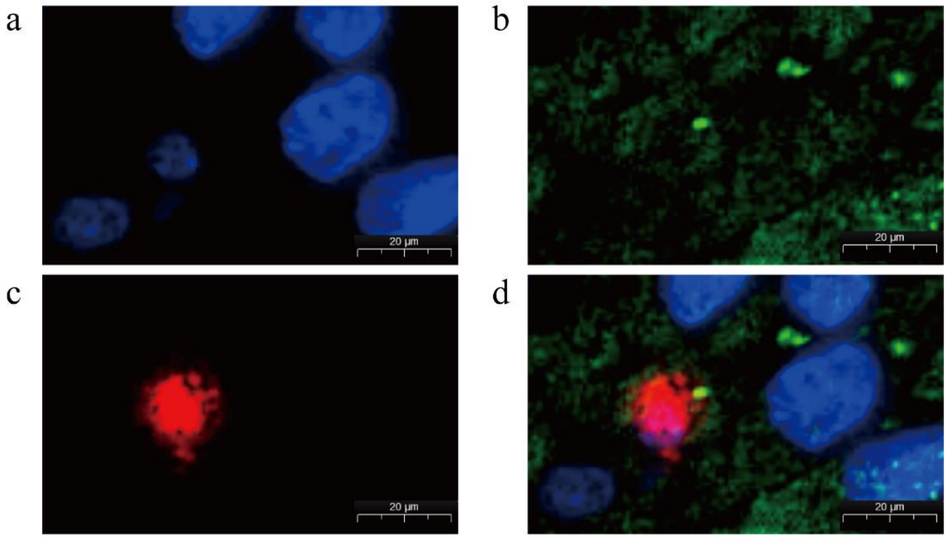

Supplementary Fig. 3 a DAPI, **b** PD-1, **c** chymase (a marker of mast cell), **d** merge of section of mouse B16-F10 tumor in 62×.

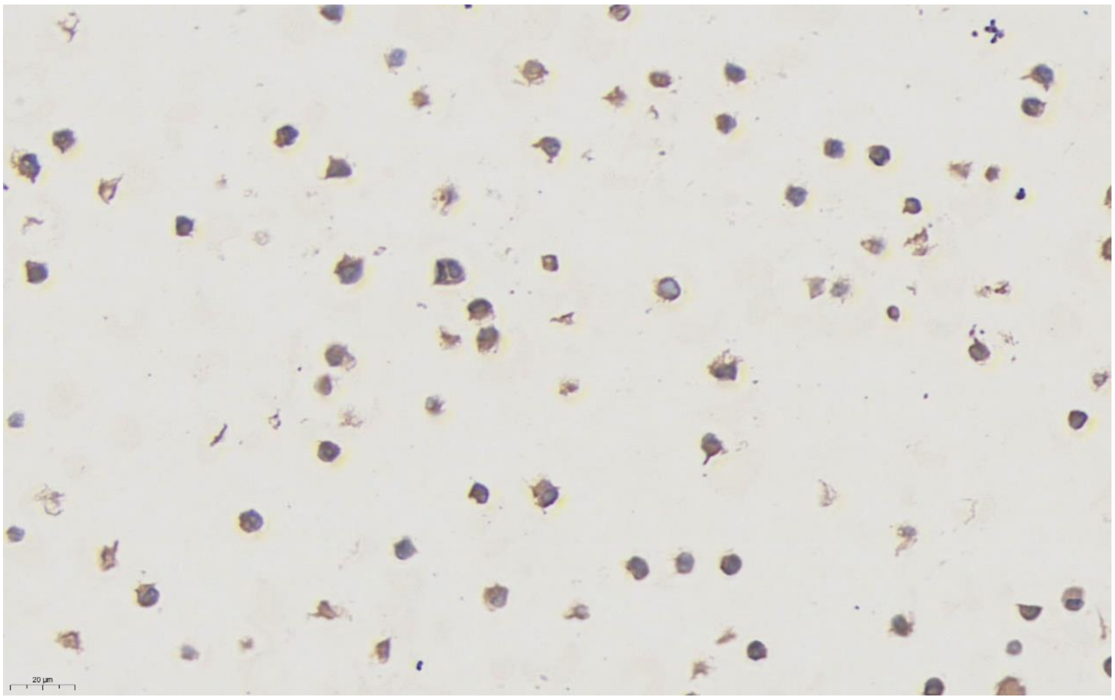

Supplementary Fig. 4 FcεRI of bone marrow-derived mast cells (BMMC). Brown FcεRI, 62×.

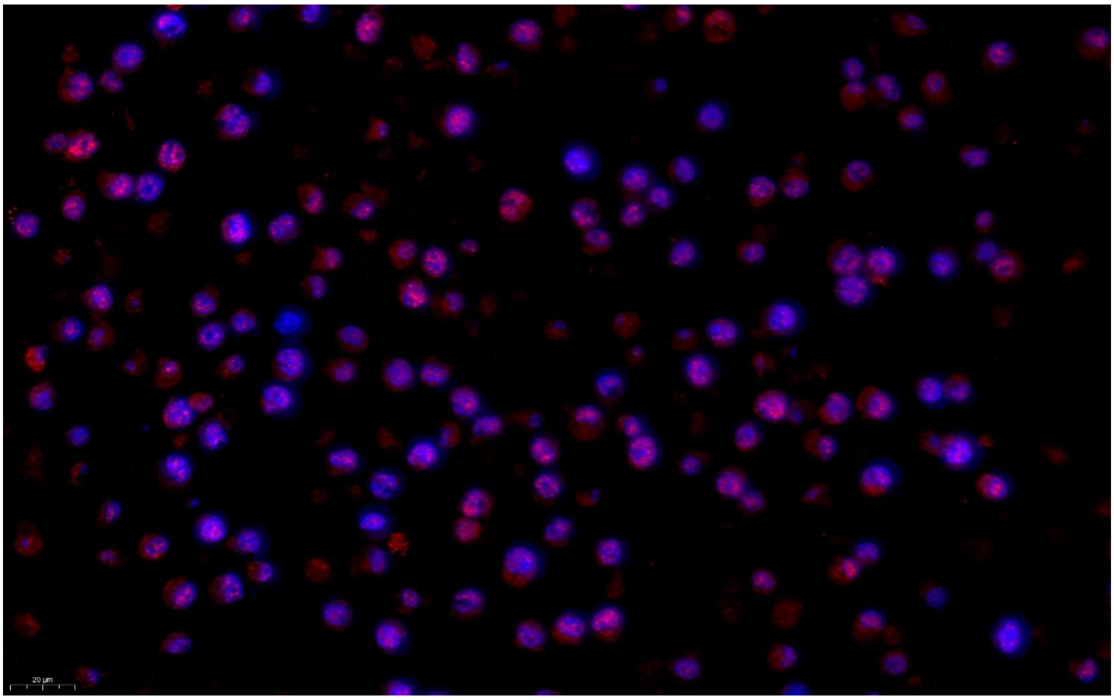

Supplementary Fig. 5 C-kit of bone marrow-derived mast cells (BMMC). Red c-kit, blue DAPI, 62×.

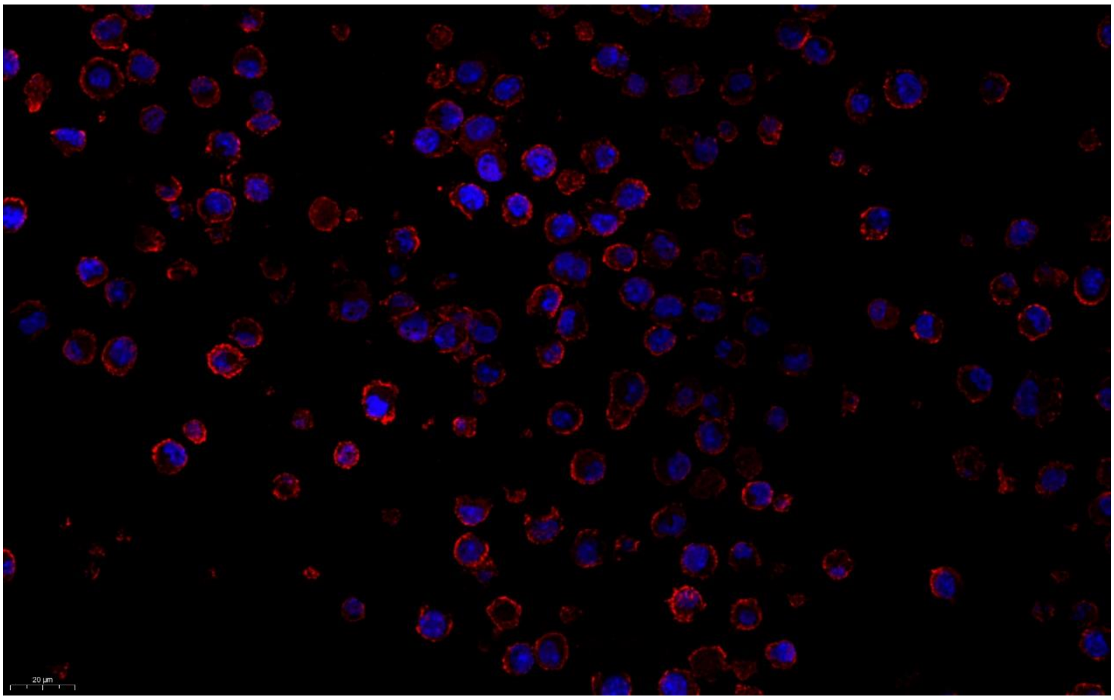

Supplementary Fig. 6 PD-1 of bone marrow-derived mast cells (BMMC). Red PD-1, blue DAPI, 62 ×

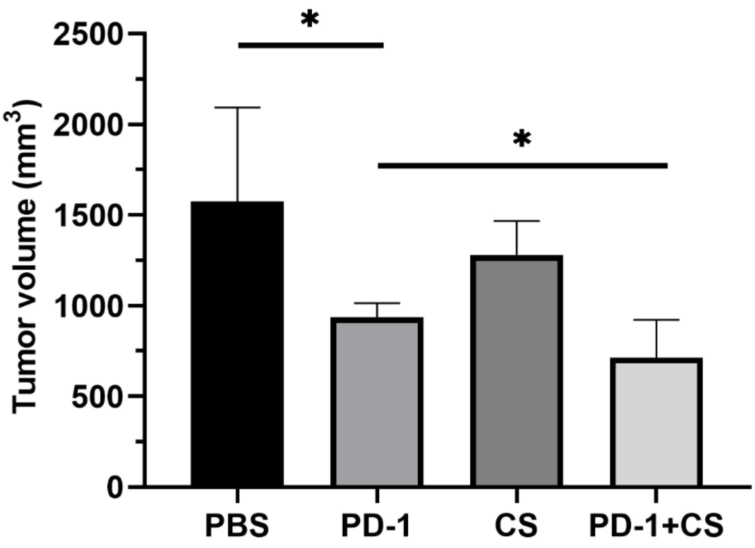

Supplementary Fig. 7 analysis of the sizes of the real tumors taken postmortem.

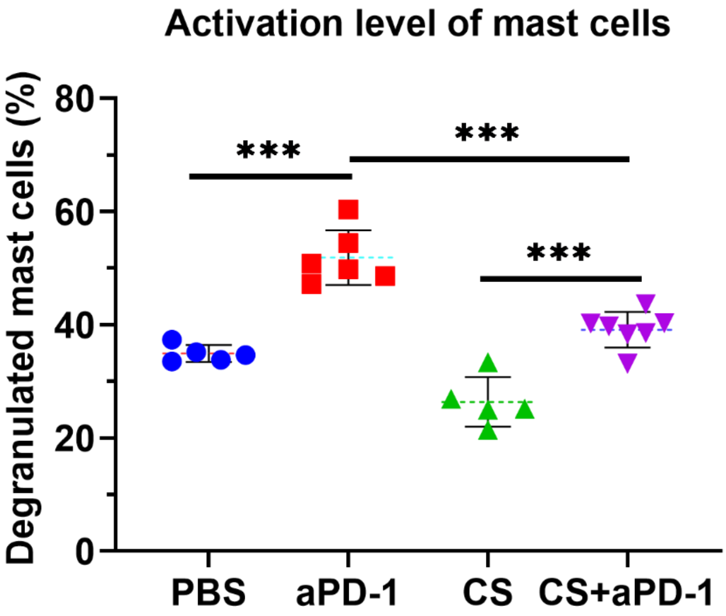

Supplementary Fig. 8 The relative fraction of degranulated mast cells in different treatment groups.
